# ASAP: Amplification, sequencing & annotation of plastomes

**DOI:** 10.1186/1471-2164-6-176

**Published:** 2005-12-07

**Authors:** Amit Dhingra, Kevin M Folta

**Affiliations:** 1 Horticultural Sciences Department, University of Florida, Gainesville, FL 32611, USA

## Abstract

**Background:**

Availability of DNA sequence information is vital for pursuing structural, functional and comparative genomics studies in plastids. Traditionally, the first step in mining the valuable information within a chloroplast genome requires sequencing a chloroplast plasmid library or BAC clones. These activities involve complicated preparatory procedures like chloroplast DNA isolation or identification of the appropriate BAC clones to be sequenced. Rolling circle amplification (RCA) is being used currently to amplify the chloroplast genome from purified chloroplast DNA and the resulting products are sheared and cloned prior to sequencing. Herein we present a universal high-throughput, rapid PCR-based technique to amplify, sequence and assemble plastid genome sequence from diverse species in a short time and at reasonable cost from total plant DNA, using the large inverted repeat region from strawberry and peach as proof of concept. The method exploits the highly conserved coding regions or intergenic regions of plastid genes. Using an informatics approach, chloroplast DNA sequence information from 5 available eudicot plastomes was aligned to identify the most conserved regions. Cognate primer pairs were then designed to generate ~1 – 1.2 kb overlapping amplicons from the inverted repeat region in 14 diverse genera.

**Results:**

100% coverage of the inverted repeat region was obtained from Arabidopsis, tobacco, orange, strawberry, peach, lettuce, tomato and *Amaranthus*. Over 80% coverage was obtained from distant species, including *Ginkgo*, loblolly pine and *Equisetum*. Sequence from the inverted repeat region of strawberry and peach plastome was obtained, annotated and analyzed. Additionally, a polymorphic region identified from gel electrophoresis was sequenced from tomato and *Amaranthus*. Sequence analysis revealed large deletions in these species relative to tobacco plastome thus exhibiting the utility of this method for structural and comparative genomics studies.

**Conclusion:**

This simple, inexpensive method now allows immediate access to plastid sequence, increasing experimental throughput and serving generally as a universal platform for plastid genome characterization. The method applies well to whole genome studies and speeds assessment of variability across species, making it a useful tool in plastid structural genomics.

## Background

Chloroplast DNA (cpDNA) represents an extranuclear capsule of genetic information, encoding essential structural and enzymatic proteins of the organelle. This satellite genome contains a wealth of information that has been shaped by speciation, rendering it a rich resource to trace evolutionary relationships between photosynthetic taxa [1]. Genetic manipulation of the chloroplast genome can transform the chloroplast into a bioreactor, allowing large-scale production of proteins vital to agriculture or pharmacology [2,3]. Maternal inheritance of plastid genome in most species ensures gene containment in genetically modified plants making it an attractive alternative for the integration of foreign genes [4,5].

Genomic and phylogenetic studies and efficient genetic modification begin with the base material of plastid DNA sequence. Despite their relatively small size, few plastid genomes have been fully sequenced, thus limiting comparative genomics studies across the species. Complete sequence coverage has been resolved for only 13 species representing model and crop plants, namely, *Arabidopsis thaliana *[6], *Atropa belladonna *[7], *Medicago truncatula *(GenBank: NC_003119), *Oryza sativa *[8], *Spinacea oleracea *[9], *Nicotiana tabacum *[10], *Triticum aestivum *[11], *Lotus corniculatus *[12], *Zea mays *[13], *Panax ginseng *[14], *Cucumis sativus *(GenBank: NC_007144), *Glycine max *[15] and *Saccharum officinarum *[16]. Comparison of a small set of representative coding or intergenic sequences derived from a large number of species has been used to perform phylogenetic studies [17], but there are many unresolved phylogenetic questions [18]. Use of complete genome data is another emerging approach in plant phylogenetics [19]. There are at least three federally-sponsored, multi-institution endeavors underway to sequence about 200 plastid genomes from plants [20-22].

There are a number of challenges to rapid access to chloroplast DNA sequence from a given species. Typical sequencing efforts begin with construction of chloroplast plasmid or other genomic libraries. Construction of such resources requires isolation of pure plastid DNA, which may be troublesome in some species. Even shotgun sequencing to appreciable coverage is considerably expensive and time consuming. From the standpoint of method and cost, the generation of plastid sequence data is considerable and a potential hindrance to productive data mining and engineering efforts. One recent report describes a sophisticated methodology using FACS (fluorescence-assisted cell sorting) and RCA (rolling circle amplification) for sequencing a plastid genome [23]. The degree of technological sophistication is inversely proportional to its wider applicability due to the prohibitive costs associated with it, slowing chloroplast genomics studies. These large-scale studies are expensive and prohibit testing of additional related species or ecotypes that may be informative. Additionally, smaller research programs do not necessarily have access to these tools and techniques. Most importantly some rare and/or difficult-to-obtain taxa that are not amenable to large-scale chloroplast DNA extraction can not be analyzed by the existing methodology. While downstream processing of sequence information is highly streamlined due to the presence of freely available tools on the World Wide Web, such as DOGMA [24,25], the lack of cost-effective innovation in rapid sequence acquisition has restricted plastid informatics studies.

To address these issues, we exploited the fact that chloroplast genomes are extremely well conserved in size, gene arrangement, and coding sequence, at least within major subgroups of the plant kingdom [26,27]. We formulated the hypothesis that conserved islands of cpDNA sequence could serve as universal anchors to generate overlapping PCR products comprised of conserved coding regions, and adjacent polymorphic intergenic regions. The resulting amplicons could then be sequenced, assembled, annotated and analyzed. This approach exploits the high degree of sequence conservation and general synteny within discrete portions of chloroplast genome. In this report this powerful technique has been applied to the large inverted repeat (IR) region of strawberry (*Fragaria *× *ananassa*) and peach (*Prunus persica*). The entire ~30 kb region was amplified from total DNA, sequenced, annotated and submitted to public databases in several days for a fraction of the cost of traditional or other recently published approaches [28]. To further validate this application, corresponding regions were amplified from a series of other eudicots and a monocot of agricultural importance as well as two gymnosperms (*Pinus *and the distant vascular plant *Gingko*) and a pteridophyte (*Equisetum*). This universal method represents a rapid, inexpensive means to obtain complete coverage of many higher plant plastid genome regions, and even substantial coverage from distant genera. The sequence information generated form this method can hasten phylogenetic and genomics studies and also help in identification of regulatory elements necessary for design of transformation vectors for the manipulation of chloroplast genomes of new species.

## Results

**Figure 1:**
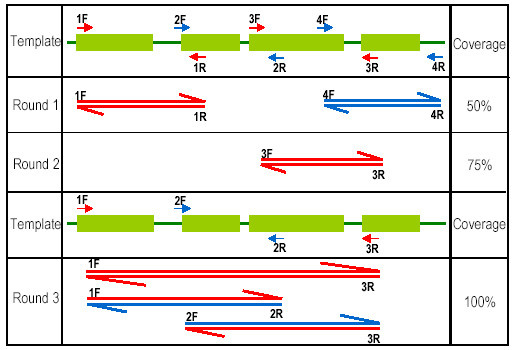
**Schematic representation of the ASAP approach**. Three rounds of PCR allow for 100% coverage of a given region. F and R suffix to the numbers represent forward and reverse universal primers.

**Figure 2:**
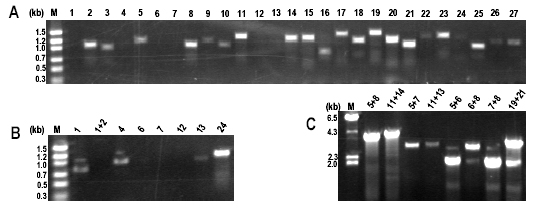
**ASAP profile from *Fragaria *× *ananassa***. A. Round 1 touchdown PCR. B. Round 2 touchdown PCR and C. Round 3 touchdown extension PCR.

In order to expand ongoing Rosaceae genomics studies [29], the original goal of this work was to use existing informatics resources to devise a PCR-based strategy to obtain plastid DNA sequence for cultivated strawberry and peach. This information would assist in identifying indel (insertion/deletion) polymorphisms or SNPs (single nucleotide polymorphisms) that could serve as an additional tool for phylogenetic analysis [30] and also allow the design of vectors useful for strawberry plastid engineering. A schematic explanation of the technique is shown in Figure 1. If a given primer pair fails to generate an amplicon in initial PCR trials, the forward primer can then be paired with the reverse primer from the next primer pair to obtain coverage of that region. Figure 2 demonstrates proof-of-concept, as universal primer set derived from the IR of five sequenced eudicots is sufficient to amplify the corresponding ~30 Kb region in commercial strawberry. The 27 primer pairs generate amplicons spanning this region. The corresponding PCR products were sequenced, and the sequence was immediately deposited to public databases. Here we proceeded from computational analyses to finished strawberry and peach IR sequence in one week for <$500.

**Figure 3:**
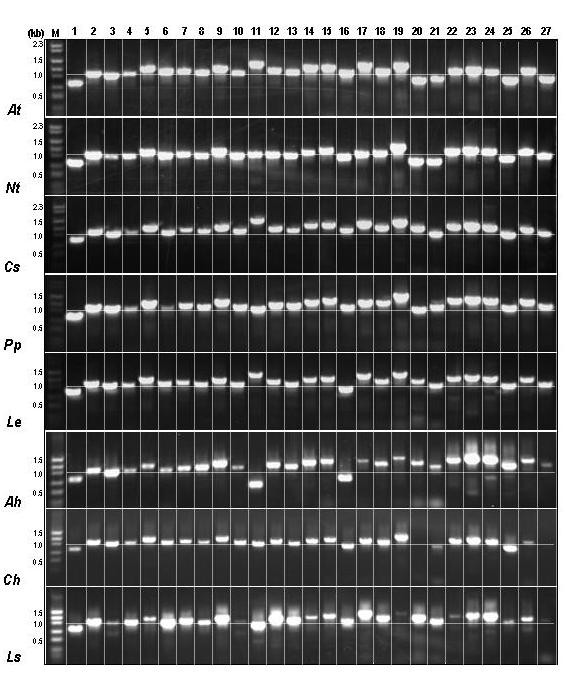
**Composite ASAP PCR profiles from 8 plant species**. ***At ***– *Arabidopsis thaliana*, ***Nt ***– *Nicotiana tabacum*, ***Cs ***– *Citrus sinensis*, ***Pp ***– *Prunus persica*, ***Le ***– *Lycopersicon esculentum*, ***Ah ***– *Amaranthus hypochondriacus*, ***Ch ***– *Coleus hybrida and ****Ls ***– *Lactuca sativa*. Horizontal lines across each species indicate 1 kb size. Vertical columns indicate the amplicons generated from a given primer pair in the 8 plant species.

Since the method proved useful in strawberry and peach, its applicability across plant species was assessed. Total genomic DNA was derived from 13 diverse plant species and subjected to the ASAP protocol using the primers listed in Table 1 and the conditions stated in Table 2. The ASAP primer set effectively generated expected amplicons from all eudicot species tested. Expected results were obtained in *Nicotiana *and *Arabidopsis*. Complete coverage was obtained with the first round of PCR and the amplicon sizes were consistent with predictions (Table 1). Comparison of agarose gel electrophoresis profiles from the IR region revealed clearly discernible amplified fragment length polymorphisms (AFLPs) in the regions amplified with primer pairs 11, 17 and 27 (Figure 3). The profiles for these two species are in complete agreement with the calculated sizes.

**Table 1:** ASAP PCR primers. Primer sequences, annealing site and the relative position in tobacco (Nt), Arabidopsis (At) and maize (Zm) are presented, along with the anticipated amplicon size. * represent the primers with low sequence similarity in maize.

**Primer**	**Gene (Nicotiana)**	**Sequence 5' to 3'**	**Position in Nt**	**Size**	**Position in At**	**Size**	**Position in Zm**	**Size**
**IRB1F**	Upstream of JLB	ggatttttttttagtgaacgtgtcac	86657–86682	804	84258–84283	812	82644–82669*	1001
**IRB1R**	Intron rpl2	aagtatcgacgtaatttcatagagtc	87436–87461		85045–85070		83622–83647	
**IRB2F**	Intron rpl2	catctggcttatgttcttcatgtagc	87397–87422	1048	85006–85031	1049	83583–83608	1043
**IRB2R**	rpl23 3'end	caactaggacagaaataaagcattgg	88420–88445		86030–86055		84601–84626	
**IRB3F**	rpl23 3'end	atacgtctgtaatgcattgtatgtcc	88310–88335	1001	85920–85945	980	84491–84516	
**IRB3R**	YCF2/ORF2280 5'	gaagatacaggagcgaaacaatcaac	89285–89311		86875–86900		Deleted	
**IRB4F**	YCF2/ORF2280	aagaaaaaatctctatttgatagggc	89181–89206	1031	86770–86795	1037	Deleted	
**IRB4R**	YCF2/ORF2280	tttcgttccgtttgaagaaaggaagg	90212–90187		87782–87807		Deleted	
**IRB5F**	YCF2/ORF2280	ggattccattagtaatgaggattcgg	90096–90121	1162	87685–87710	1171	Deleted	
**IRB5R**	YCF2/ORF2280	gaggctcgaaccatttcttctgactc	91233–91258		88831–88856		Deleted	
**IRB6F**	YCF2/ORF2280	cttcgaatatggaattcaaagggatc	91131–91156	1042	88729–88754	1081	Deleted	
**IRB6R**	YCF2/ORF2280	tgaatatgttagatacctgtgactcg	92148–92173		89785–89810		Deleted	
**IRB7F**	YCF2/ORF2280	acaattcctcaatatcttgttcattc	92049–92074	1091	89680–89705	1088	Deleted	
**IRB7R**	YCF2/ORF2280	tcttctagagaatctcctaattgttc	93115–93140		90743–90768		Deleted	
**IRB8F**	YCF2/ORF2280	gaaaaggtcaaatctttgatgattcc	92995–93020	1041	90623–90648	1035	Deleted	
**IRB8R**	YCF2/ORF2280	tttccggcatcatatccatagttagc	94011–94036		91633–91658		Deleted	
**IRB9F**	YCF2/ORF2280	ctgaacaagttcctggataacaagcc	93853–93878	1169	91493–91518	1151	Deleted	
**IRB9R**	YCF2/ORF2280	aaatctctgatcaaggatagaacaag	94997–95022		92619–92644		Deleted	
**IRB10F**	YCF2/ORF2280	gatctagttcatggcctattagaagt	94849–94874	1025	92471–92496	1021	86077–86102	1138
**IRB10R**	ORF 87/YCF 15	taacatattcttccatggagctaagg	93849–95874		93475–93492		87190–87215*	
**IRB11F**	YCF2/ORF2280 3'	cggatgaaatgaaaattggattcatg	95669–95724	1094	93330–93355	1319	No similarity	
**IRB11R**	ORF 79	aatcggacctgctttttacatatctc	96739–96763		94624–94649		No similarity	
**IRB12F**	ORF 79	ccaattgcttcgatttgaattatccg	96626–96644	1061	94483–94508	1101	88794–88819	1079
**IRB12R**	ndhB 3' exon	tggaaatcctagctattcttagcatg	97662–97687		95559–95584		89848–89873	
**IRB13F**	ndhB 3' exon	attccaataattacatatccgatttg	97567–97592	1043	95464–95489	1049	89753–89778	1068
**IRB13R**	ndhB 5' exon	cttatcaatacacaaatgtataactc	98585–98610		96488–96513		90796–90821	
**IRB14F**	ndhB 5' exon	tacgtcaggagtccattgatgagaag	98494–98519	1171	96397–96422	1206	90705–90730	1192
**IRB14R**	rps 7 5'end	aatatggctttcaaattaagttccga	99640–99665		97578–97603		91872–91897	
**IRB15F**	rps 7 5'end	gtgcaaaagctctatttgcctctgcc	99551–99576	1230	97489–97514	1231	91783–91808	1245
**IRB15R**	rps12 exon 2	tcactgcttatatacccggtattggc	100754–100781		98695–98720		93003–93028	
**IRB16F**	rps12 exon 2	tcctcgaacaatgtgatatctcacac	100699–100694	968	98608–98633	1079	92916–92941	859
**IRB16R**	Spacer	caacataggtcatcgaaaggatctcg	101642–101667		99662–99687		93750–93775	
**IRB17F**	Spacer	gtgtgagcttatccatgcggttatgc	101554–101581	1116	99576–99601	1345	93669–93694	1339
**IRB17R**	rrn16 start	gcttcatattcgcccggagttcgctc	102645–102670		100896–100921		95043–95068*	
**IRB18F**	trnV 3' end	aagtcatcagttcgagcctgattatc	102504–102529	1117	100751–100776	1122	94901–94926	1121
**IRB18R**	rrn16 start	tgagtttcattcttgcgaacgtactc	103596–103621		101848–101873		95997–96022	
**IRB19F**	rrn16	cgacactgacactgagagacgaaagc	103452–103477	1343	101704–101729	1341	95853–95878	1347
**IRB19R**	trnI Intron/OriA	atcgaaagttggatctacattggatc	104770–104795		103020–103045		97175–97200	
**IRB20F**	trnI start	gggctattagctcagtggtagagcgc	104551–104576	877	102801–102826	898	96953–96978	1119
**IRB20R**	trnA start	caagagcggagctctaccaactgagc	105403–105428		103674–103699		98047–98072	
**IRB21F**	trnI end	gaggtctctggttcaagtccaggatg	105229–105324	941	103571–103596	962	97943–97968	968
**IRB21R**	trnA end	ataagcggactcgaaccgctgacatc	106145–106170		104508–104533		98886–98911	
**IRB22F**	trnA intron	agattttgagaagagttgctctttgg	106003–106028	1190	104367–104392	1188	98743–98768	1193
**IRB22R**	23S	tagatgtccagtcaactgctgcgcct	107168–107193		105530–105555		99911–99936	
**IRB23F**	23S	gaaactaagtggaggtccgaaccgac	107053–107079	1225	105416–105441	1224	99797–99822	1291
**IRB23R**	23S	cgctaccttaggaccgttatagttac	108253–108278		106615–106640		101063–101088	
**IRB24F**	23S	ggtctccgcaaagtcgtaagaccatg	108131–108156	1172	106493–106518	1169	100941–100966	1156
**IRB24R**	4.5S	acatcactgcacttccacttgacacc	109278–109303		107637–107662		102072–102097	
**IRB25F**	23S end	ctgctgaaagcatctaagtagtaagc	109089–109115	963	107451–107476	929	101897–101922	920
**IRB25R**	trnR end	ggttgtgggcgaggagggattcgaac	110027–110052		108355–108380		102792–102817	
**IRB26F**	Spacer	aaatggctggggagaggaaaggttcc	109905–109930	1182	108231–108256	1232	No similarity	
**IRB26R**	ORF350	attatcttcatgcataaggatactag	111062–111087		109438–109463		No similarity	
**IRB27F**	Spacer	tggctctatttcattatattccatcc	110844–110869	1036	109225–109250	986	No similarity	
**IRB27R**	ORF 350	agtggatccctcttgttcctgtttag	111855–111880		110186–110211		No similarity	

**Table 2:** ASAP PCR conditions. The thermalcycler parameters used to generate ASAP amplicons in successive rounds of PCR are presented.

**ASAP PCR conditions**		
**1. Touchdown PCR**	Temperature (Centigrade)	Duration (Minutes)
Denaturation	94	4

10 Cycles		
Denaturation	94	0:40
Annealing	55 – 0.5/cycle	0:40
Extension	72	0:40

25 Cycles		
Denaturation	94	0:40
Annealing	50	0:40
Extension	72	0:40

Final extension	72	7

**2. Touchdown PCR**		

Denaturation	94	4

10 Cycles		
Denaturation	94	0:40
Annealing	52 – 0.5/cycle	0:40
Extension	72	0:40

25 Cycles		
Denaturation	94	0:40
Annealing	47	0:40
Extension	72	0:40

Final extension	72	7

**3. Touchdown – extension PCR**		

Denaturation	94	4

10 Cycles		
Denaturation	94	0:40
Annealing	52 – 0.5/cycle	0:40
Extension	72	1:00 + .05/cycle

25 Cycles		
Denaturation	94	0:40
Annealing	47	0:40
Extension	72	1:30 + 0.05/cycle

Final extension	72	10

**Figure 4:**
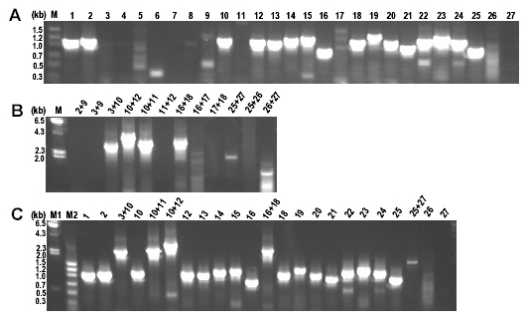
***Zea mays *ASAP PCR profiles**. A. Round 1 and 2 touchdown PCR. B. Round 3 touchdown extension PCR and C. Composite profile from A and B.

The maize plastid genome lacks the *ycf*2 open reading frame in the IR region, therefore primer pairs 3 to 9 failed to produce any amplicons, as anticipated (Figure 4A). Similarly, primer pairs 11, 26 and 27 did not produce any amplicons. Using bl2Seq program the maize IR region was compared with the associated primers and no significant sequence similarity was found between them. Interestingly, one primer each in primer pairs 1 and 10 had very low sequence similarity and yet the amplicons were obtained. Using three rounds of PCR (Table 2) 100% coverage was obtained even in the monocot plastome (Figure 4C), indicating the applicability of eudicot-based primer designs to this taxonomic group. Absence of amplicons from primer pair 26 and 27 could be due to the fact that the primers annealed in the spacer region which could be unique to the eudicots. The results of the reactions are presented in both Table 3 and Figure 3. Table 3 presents the conditions required to produce the amplified regions from individual species, whether the products were obtained from PCR round 1, round 2 or round 3. Figure 3 shows the complete array of amplified products corresponding to the amplification conditions presented in Table 2.

**Table 3:** ASAP coverage. Percentage coverage of the IR B region using ASAP method in 10 different genera and unique features of 4 diverse genera used in the study.

**Percentage coverage of the IR region using ASAP**					
**Species**	**Family**	**Round 1 (%)**	**Round 2 (%)**	**Round 3 (%)**	**Final**
*Arabidopsis thaliana*	Crucifereae	100			100
*Nicotiana tabacum*	Solanaceae	100			100
*Citrus sinensis*	Rutaceae	100			100
*Lycopersicon esculentum*	Solanaceae	59	100		100
*Prunus persica*	Rosaceae	7.4	100		100
*Amaranthus*	Amaranthaceae	81	100		100
*Lactuca sativa*	Asteraceae	63	100		100
*Fragaria *× *ananassa*	Rosaceae	74	89	100	100
*Coleus *× *hybrida*	Lamiaceae	85	92	N/A	92
*Zea mays*	Poaceae	40	80	100	100
					
**Unique sps**					
*Pisum sativum*	Fabaceae	No Inverted Repeat			
*Ginkgo biloba*	Ginkgoaceae	Small inverted repeat of 17 kb			
*Pinus taeda*	Pinaceae	Gymnosperm			
*Equisetum hyemale*	Equisetaceae	Pteridophyte			

### *Fragaria *and *Prunus *(Rosaceae)

Complete coverage of the plastid IR region from *Fragaria *was obtained after proceeding through three rounds of ASAP PCR (Table 2) with the 27 pairs of primers (Figure 2). These amplicons were generated using *Pfu *Turbo DNA polymerase (Stratagene Inc., Carlsbad, CA) in order to minimize potential errors generated during PCR reactions. These amplicons were directly sequenced in a 96-well format. The sequence was assembled and annotated as described in Methods.

Interestingly, in *Prunus *complete coverage was obtained with Round 2 PCR. Sequence comparison with *Fragaria *revealed that *Prunus *and *Fragaria *share considerable sequence similarity in the IR region as expected being from the same phylogenetic group. This is another demonstration of the utility of this technique where two members of the same taxonomic group were sequenced and compared in a very short time frame and in a cost-effective manner.

### Other eudicots and identification of a variable region

**Figure 5:**
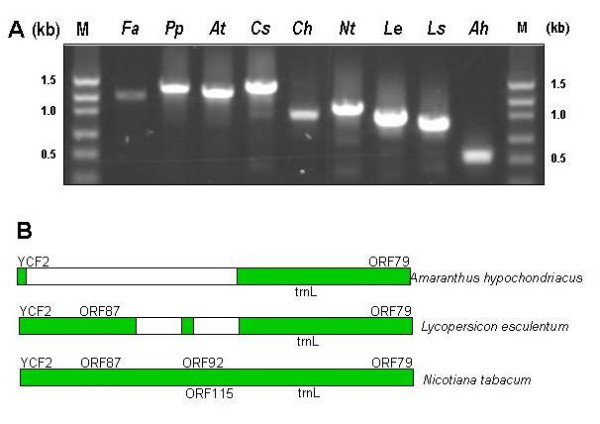
**A. PCR amplicons generated from primer pair 11 in 9 plant species**. ***Fa ***– *Fragaria *× *ananassa*/Rosaceae, ***Pp ***– *Prunus persica*/Rosaceae, ***At ***– *Arabidopsis thaliana*/Crucifereae, ***Cs ***– *Citrus sinensis*/Rutaceae, ***Ch ***– *Coleus hybrida*/Lamiaceae, ***Nt ***– *Nicotiana tabacum*/Solanaceae, ***Le ***– *Lycopersicon esculentum*/Solanaceae, ***Ls ***– *Lactuca sativa*/Asteraceae, ***Ah ***– *Amaranthus hypochondriacus*/Caryophyllaceae. **B**. Schematic representation of the polymorphism between the amplicons generated from primer pair 11 in tobacco, tomato and amaranthus.

The ASAP protocol was attempted in other eudicot species for which plastid genome sequence has not been reported. In *Citrus *and *Lycopersicon *complete coverage was obtained after Round 1 PCR and for the remaining species almost 99 – 100% coverage was obtained using Round 2 PCR conditions. Electrophoresis profiles revealed highly discernible AFLPs amongst different plant species. The most consistently variable region was represented by primer pair 11. In tobacco this amplicon represents sequences for *orf*87/*ycf*15, *orf*92, *orf*115, *trn*L and *orf*79. Gel electrophoresis profiles of amplicons generated from this primer pair revealed a great range of variability across all species tested (Figure 5A). AFLPs were discernable by gel electrophoresis between two solanaceous species, *Nicotiana *and *Lycopersicon*. Sequencing and alignment of this region from two members of Solanaceae, tobacco and tomato revealed a 95% – 98% sequence similarity in the aligning sequences. Tomato had two deletions in the region coding for *orf*92 and *ycf*15 in tobacco, which reconciles the smaller amplicon size. On the other extreme is the representative member of Caryophyllaceae, *Amaranthus*, where sequencing and subsequent alignments with tobacco revealed absence of ORFs between *ycf*2 and *orf*92 – *trn*L-CAA region (Figure 5B). Thus the ASAP method provides the advantage of analyzing a large region from a number of species and identifying a highly variable region at the same time.

### Distant species

**Figure 6:**
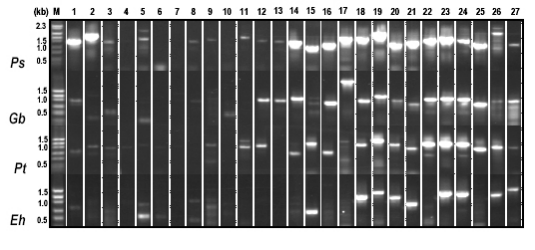
**Composite ASAP PCR profiles from 4 unique plant species**. *Ps *– *Pisum sativum*, *Gb *– *Gingko biloba*, *Pt *– *Pinus taeda*, *Eh *– *Equisetum hyemale*.

To test the limits of this methodology, the same 27 pairs of primers were used against total DNA from *Pisum sativum*, *Ginkgo biloba*, *Pinus taeda *and *Equisetum hyemale*. These species represent a unique member of Fabaceae (*Pisum *– largest deletion resulting in removal of the rRNA cluster; has only one IR), an ancient and contemporary gymnosperm, and a pteridophyte. The primer pairs designed for eudicot plastid genomes were able to amplify the regions corresponding to primer pairs 14 to 25 in *Pisum*. In tobacco these primer pairs amplify the 98494 – 110052 nt region of the IR that includes the *rrn *operon. The bl2Seq program was used to determine the sequence similarity between the 27 primer pairs and the *Pisum *chloroplast genome sequence (Kindly provided by John Gray, John Innes Institute, UK). The observed amplicon patterns are consistent with what is anticipated from the sequence data. Primer pairs that failed to generate an amplicon do not share a significant sequence similarity with the *Pisum *plastid genome sequence (Figure 6). In the two gymnosperms, similar amplicon patterns were generated from the *rrn *operon region. Again in the pteridophyte only the primer pairs corresponding to the *rrn *operon produced an amplicon. The *Equisetum *chloroplast genome does possess *ycf*2 gene but comparative sequence analysis with higher plant *ycf*2 revealed no significant sequence similarity. Interestingly the amino acid sequence similarity was almost 94% (data not shown).

## Discussion

Chloroplast sequencing efforts of model photosynthetic organisms have provided a wealth of information detailing structural features and plant phylogeny, as well as a basis for manipulation of the plastid genome in the interest of bioengineering. Current molecular phylogenetic studies are carried out using large or complete plastid genome sequences or small coding or intergenic sequences and the Amplification, Sequencing, and Annotation of Plastomes (ASAP) method caters to both approaches. Rapid generation of plastid sequence information is necessary as it can facilitate better design of plastid transformation vectors [15]. One of the factors in successful engineering of cotton and carrot plastomes was the use of species-specific flanking sequences in the transformation vectors [31,32].

The simple yet powerful ASAP technique described herein expands the capacity for any laboratory to dissect the chloroplast genome at the informatics level with a basic set of available resources. The obvious limitation of ASAP method is that some plastid genomes have undergone extensive rearrangements [33]. Therefore, this method complements other strategies [28] for obtaining plastid genome sequences and provides a convenient platform for plastomes that share a considerable level of synteny. A long range PCR based approach was used earlier to sequence the *Amborella trichopoda *plastid genome [34], requiring nebulization of large amplicons and cloning prior to sequencing. In contrast, the ASAP method performs the dual task of generating small amplicons for direct sequencing and the electrophoresis profiles provide structural genomics information. Direct sequencing of the PCR products limits incorrect base calls resulting from amplification or sequencing errors, as large pools of products are sequenced and even early PCR errors will not be scored incorrectly. In a worst-case scenario a base call at a given position will be unclear, requiring re-amplification and re-sequencing of the amplicon. This approach allows the implementation of non-proofreading, highly processive polymerases that limit costs yet generate substantial quantities of template for downstream analyses.

ASAP also allows description and characterization of the frequent islands of high sequence identity present within the coding regions of sequenced genomes. Using local identities found within the cpDNA sequences of five sequenced eudicot plant species, primer pairs were designed to produce overlapping amplicons that bracket sequences of plastid genes and the rich sequence variability resident to their adjacent intergenic regions. The ASAP method was herein shown to generate representative regions of 10 diverse plant species to almost 100% coverage. Even the *rrn *region was also amplified from four divergent genomes studied, such as *Pisum*, *Equisetum*, *Pinus *and *Ginkgo*, with expected efficiency. The success of the method suggests it an excellent first step in the analysis of any novel plastid genome. The ASAP method may be the only practical approach for some rare and/or difficult-to-obtain taxa that are not amenable to chloroplast DNA extraction.

One caveat of this technique is that plastid sequences are not confined to the chloroplast. Plastid DNA sequences are represented in both mitochondrial and nuclear genomes, and may serve as templates for the amplicons generated with the 27 primer pairs. The high copy number of plastomes in green leaf derived total DNA and long primers (26 bp each) used in this approach should preferentially amplify chloroplast sequences. Additionally, nuclear integrated plastid sequences are continuously shuffled and eliminated [35], making them less likely to be incorrectly amplified via this approach. However, with the design of specific primers this technique could be extended to plastid genomes of distant phylogenetic groups or mitochondrial genomes in species where high level of synteny is present.

## Conclusion

The ASAP method represents a rapid means to generate a large amount of plastid genome information from simple PCR steps to facilitate bioinformatic dissection and functional genomic studies. The products generated spotlight AFLPs that serve as low-resolution beacons to report regions of high diversity, such as the hypervariable Region 11 (Figure 5). These regions may be particularly meaningful for phylogenetic analyses. In this capacity the ASAP methods may facilitate studies of hypervariable specific regions of the plastid genome, helping to quickly identify areas of likely importance, bypassing the necessity to sequence an entire genome as a first step. The ASAP method eliminates cloning steps, thus negating the need to identify and trim plasmid sequences before assembly. Another powerful facet of the technique is the potential to generate plastid fragment fingerprint for any species. AFLPs produced may be immediately comparable to those produced by other genomes, revealing deviations in otherwise conserved sequence, thus informing of structural-genomic variation. Even the absence of products in related species may be extremely informative.

To summarize, the ASAP method has the following advantages: The method is ideal for laboratories or programs with limited resources interested in obtaining chloroplast DNA sequence information for their particular research interest. ASAP method may be the only practical approach to obtain chloroplast DNA sequence from rare or small plant samples. This method provides a fingerprint of a given chloroplast region, which can be readily compared amongst different genera and give information of structural variability even without the sequence information. Another practical application of this approach is the use of PCR amplicons generated by ASAP method for construction of chloroplast genome microarrays from a given plant species.

Specific to this report, the method was used to amplify and sequence the large IR region from octoploid strawberry and peach. This ~30 kbp region from strawberry was amplified in 33 PCR reactions and then sequenced. The PCR fragments were generated in three thermalcycler runs over two days, and the entire process, from leaf to data on the server, was performed in under a week for under $500. With this minimal time and capital investment roughly 25% of the chloroplast genome has been deciphered; bidirectionally and with complete coverage. The entire process is now being scaled up to sequence an entire plastid genome within the context of a 96-well plate. In this system 1.2 to 1.5 kb amplicons may be produced and sequenced from this standard format. The common format also lends itself to robotic manipulation, and it is exciting to speculate that a single set of 96 primer pairs may be matched to a high-throughput robotic amplification and sequencing system to generate plastid DNA sequence at the rate of a plastid genome per day. It is our hope that this methodology will hasten study of chloroplast sequences, especially those from unusual organisms or those not considered worthy of large investment.

## Methods

### Primer design

The cpDNA sequences of five eudicot plant species namely *Nicotiana tabacum *(NC_001879), *Arabidopsis thaliana *(NC_000932), *Atropa belladonna *(NC_004561), *Spinacea oleracea *(NC_002202) and *Panax ginseng *(NC_006290) were aligned using ClustalW [36]. Tobacco chloroplast genome sequence was used as a reference and its coding regions were delineated in the aligned sequences. Highly conserved, putative primer sites were derived by hand parsing the aligned sequences of the IR B region. Primer candidates satisfied several criteria. A candidate primer must be resident to the coding region or conserved intergenic region and primer pairs must be spaced by ~1.0 to 1.2 kb. The primers must share 95% sequence identity among the representative plastid genomes. For universal, large-format application in simultaneous PCR reactions the primers should maintain approximately 50% GC content and a T_m _of approximately 50°C. Table 1 lists the primer sequences, annealing sites, respective position in tobacco, Arabidopsis and maize plastid genomes and the expected amplicon sizes. This set of primers, used to amplify the large IR region in this manuscript, will be supplied by the authors upon request.

### DNA preparation and primary optimization

Total plant DNA was isolated from fresh leaf tissue using the Qiagen DNeasy Kit (Qiagen Inc., Valencia, CA) according to manufacturer's instructions, except that the homogenized plant material was mixed in Buffer AP1 for 10 min on a platform vortexer for 10 min and the sample was centrifuged for 10 min at 1000 rpm to remove any debris at this stage. The supernatant was then incubated at 65°C for 10 min and from this point on the manufacturer's protocol was followed. DNA was isolated from two plant species namely *Arabidopsis thaliana *(ecotype Col-0) and tobacco (*Nicotiana tabacum*). These two species served as a positive control and a basis for detection of computationally predicted AFLPs. Upon validation of the technique with known genomes, amplification was performed on several crops of agricultural importance namely, strawberry (*Fragaria *× *ananassa*; cv. Strawberry Festival), Sweet orange (*Citrus sinensis*), lettuce (*Lactuca sativa*), peach (*Prunus persica*; cv. UF Sun), Tomato (*Lycopersicon esculentum*), coleus (*Coleus *× *hybrida*), *Amaranthus *(*Amaranthus hypochondricus*). Maize (*Zea mays*; W22) was tested as a model monocot. Since it has been sequenced, amplification discrepancies related to insertion/deletion and/or rearrangement can be anticipated and circumvented. The technique was also performed on diverse plant species to test the range of the approach. These species included *Pisum sativum *L. (Little Marvel), which does not have an IR region and has only one copy of the rRNA genes, an ancient gymnosperm (*Ginkgo biloba*), a contemporary gymnosperm loblolly pine (*Pinus taeda*), along with horsetail, (*Equisetum hyemale*), a pteridophyte which represents an ancient plastid genome that would test and define the limit of the application of the eudicot species-based primer designs reported here.

Before performing reactions with the 27 primer pairs that define the large IR, it was important to establish the amount of total DNA to use in each reaction. Variability in amplification may be introduced from several sources, namely the relative amount of chloroplast DNA to total DNA ratio and the tendency for inhibitory compounds to co-purify with DNA templates in total DNA isolation [37]. For instance, the relative cpDNA: total DNA ratio will be significantly different between Arabidopsis (haploid genome size ~140 Mbp) and *Pinus *(haploid genome size ~21658 Mbp), two organisms with massively different nuclear genome sizes. DNA preparation from some species, such as strawberry, may introduce phenolics or polysaccharides that could inhibit efficient amplification during PCR.

To accommodate both of these variables an initial reaction set is performed using the most conserved primers, those representing the 16 S *rrn *locus (primer set 19; Table 1). These primers predictably generate a fragment in all species tested, yet the yield varies considerably based on the amount of template used in the reaction. For instance, while coleus was best amplified with 1 ng per reaction, Equisetum required 10 ng per reaction (data not shown). The fidelity of the reaction is extremely dependent upon total isolated DNA concentration and the inherent cpDNA: nuclear DNA ratio in total DNA. A pilot experiment testing for the production of PCR product over 4–5 orders of magnitude must be performed to optimize conditions for subsequent reactions.

### PCR conditions for plastome amplification

Touchdown PCR was utilized to generate PCR amplicons [38]. The first set of reactions was performed using Round I conditions, conditions conducive to amplification in the species for which the primers were designed (Figure 1; Table 2). This reaction would routinely produce amplicons in 24 out of 27 reactions. Reactions that failed to produce a product were reconstituted from fresh reagents and template, and PCR is performed using Reaction II conditions (Table 2). Typically this amplification was sufficient to obtain complete coverage of the large IR in 7 of the 10 species studied (Table 3). Amplicons generated from this approach will be made feely available upon request.

If Round II conditions failed to produce a PCR product it could be assumed that sequence differences in the primer landing site are present or those sites are deleted or rearranged. In this case PCR is performed using each fragment-specific primer and primers from adjacent fragments that successfully amplified in Round I and/or Round II. The process is outlined in Figure 1.

Sequencing was performed directly from PCR products in a 96-well format at the University of Florida ICBR Core Facility using ET Terminator (Amersham Inc, Schaumburg, IL) as reported earlier [29]. A 5 μl aliquot of PCR product from a 50 μl reaction was analyzed by gel electrophoresis to verify purity and concentration. PCR products were treated with ExoSAP to remove primers and nucleotides. Each amplicon was sequenced bidirectionally using the primers used in initial amplification. Sequencing primers were added at 10 pmol/μl in a 10 μl final reaction volume. Sequences with Phred score of >20 were used for assembly. Both strands were sequenced for each fragment thus providing a 2X coverage and, 4X coverage in the overlapping regions.

### Assembly, annotation and dissemination of the sequences from *Fragaria *and *Prunus*

Sequences generated from a primer pair were first aligned using Blast 2 sequences (bl2seq) available at NCBI website [39,40]. Individual amplicons derived from this process were further assembled using CAP3 [41,42]. The sequence was then annotated using DOGMA [24,25]. Chloroplast genome sequences from the IR B region of *Fragaria *and *Prunus *were submitted to the GenBank under accession numbers FACPINVREP (*Fa*) and PPCPINVREP (*Pp*).

## List of abbreviations

ASAP: Amplification, sequencing, annotation of plastomes; ORF: Open reading frame; AFLP: Amplified fragment length polymorphism; SNP: Single nucleotide polymorphism; PCR: Polymerase chain reaction; FACS: Fluorescence-assisted cell sorting; RCA: Rolling circle amplification; DOGMA: Dual organeller genome annotator

## Authors' contributions

AD conceived of the ASAP methodology, designed primers, prepared DNA, performed all PCR/sequencing reactions, and assembled/reported all sequences. AD also prepared all figures and tables for the manuscript. KMF assisted in shaping the ASAP concept and prepared the first draft of the manuscript. Both authors contributed to the development of the final manuscript.
